# Spatial analysis of cholangiocarcinoma in Thailand from 2012 to 2021; a population-based cancer registries study

**DOI:** 10.1371/journal.pone.0311035

**Published:** 2024-12-11

**Authors:** Oraya Sahat, Supot Kamsa-ard, Apiporn Thinkhamrop Suwannatrai, Apiradee Lim, Siriporn Kamsa-ard, Surichai Bilheem, Karnchana Daoprasert, Atit Leklob, Surin Uadrang, Chalongpon Santong, Nithima Sriket, Wasan Chansaard

**Affiliations:** 1 Student of Doctor of Public Health Program, Faculty of Public Health, Khon Kaen University, Khon Kaen, Thailand; 2 Department of Epidemiology and Biostatistics, Faculty of Public Health, Khon Kaen University, Khon Kaen, Thailand; 3 Department of Parasitology, Faculty of Medicine, Khon Kaen University, Khon Kaen, Thailand; 4 Department of Science in Mathematics with Computer Science, Faculty of Science and Technology, Prince of Songkla University, Pattani Campus, Pattani, Thailand; 5 Department of Occupational Health and Safety, Sirindhorn College of Public Health, Yala, Yala, Thailand; 6 Lampang Cancer Hospital, Lampang, Thailand; 7 Lop Buri Cancer Hospital, Lop Buri, Thailand; 8 Cancer Unit, Srinagarind Hospital, Faculty of Medicine, Khon Kaen University, Khon Kaen, Thailand; 9 Surat Thani Cancer Hospital, Surat Thani, Thailand; Niigata University of Pharmacy and Medical and Life Sciences, JAPAN

## Abstract

**Background:**

Cholangiocarcinoma (CCA) is major health issue in Thailand, with high incidences in different parts of country. This study examines the association between spatial variables and CCA in Thailand.

**Methods:**

Thailand’s four population-based cancer registries provided data for this retrospective cohort analysis between January 1, 2012, and December 31, 2021. 6,379 diagnosed CCA cases were found in 554 sub-districts. Moran’s *I* and Local Indicators of Spatial Association (LISA) measured geographic dispersion and estimated sex age-standardized rates (ASRs). Multivariable log-linear regression assessed geography and CCA, calculating adjusted incidence rate ratios (IRRs) and 95% confidence interval (CI).

**Results:**

Of 6,379 CCA cases, 63.9% were male, and the mean age at diagnosis was 66.2 years (standard deviation = 11.07 years). CCA ASRs for both sexes in Thailand was 8.9 per 100,000 person-years. The northeastern region had the greatest ASR at 13.4 per 100 000 person-years. Moran’s *I* and LISA studies grouped regions by spatial variables. The association between spatial variables and CCA demonstrated that the northern region exhibited elevation (adjusted IRRs = 0.82, 95%CI: 0.78 to 0.87) and distance from water sources variable (adjusted IRRs = 0.91, 95%CI: 0.82 to 0.99). The central region elevation variable (adjusted IRRs = 0.85, 95%CI: 0.76 to 0.94). This was the distance from water sources (adjusted IRRs = 0.96, 95%CI: 0.93 to 0.99) and population density variable (adjusted IRRs = 0.94, 95%CI: 0.93 to 0.96) in the northeastern region. Population density (adjusted IRRs = 1.09, 95%CI: 1.02 to 1.15) and average temperature variable (adjusted IRRs = 1.05, 95%CI: 1.02 to 1.09) were significant in the southern region.

**Conclusion:**

Spatial variables associated with CCA indicate that ASR differs across Thailand. So environmental and climate factors can inform targeted public health strategies to address CCA in high-risk areas throughout Thailand.

## Introduction

Cholangiocarcinoma (CCA) is a cancer that develops in the bile duct lining. It is anatomically classified into two subtypes—intrahepatic (within the liver) or extrahepatic (outside the liver) [1–4]. According to a report by the World Health Organization, liver and bile duct cancer are ranked as the seventh most diagnosed cancer and the second leading cause of cancer-related deaths in 2020. Globally, there were 905,677 new cases of liver and bile duct cancer, resulting in 830,180 deaths across 11 geographically diverse countries, including North Africa, West Africa, East Asia, and Southeast Asia. In Asia, 656,992 cases of liver and bile duct cancer have been reported, with 608,898 deaths attributed to this malignancy [5].

The age-standardized rates (ASRs) of liver and bile duct cancers in Asia are the fifth highest in the world. The ASR for both sexes is 11.6 per 100,000 per year—17.3 for males and 6.2 for females. The ASRs in Southeast Asia were number one in the region, and the annual incidence of liver and bile duct cancer in males is 21.2 per 100,000, while it is 7.1 per 100,000 in females. Studies have found that Mongolia, Thailand, Cambodia, and Egypt have the highest rates of mortality due to liver and bile duct cancers [5, 6].

The incidence of liver and bile duct cancer in Thailand was 27,394 new patients, 33.8 per 100,000 person-years in males and 12.9 per 100,000 person-years in females [6]. The statistics from cancer registries nationwide were recorded in a monograph on “Cancer in Thailand from 2004 to 2018”. The incidence of liver and bile duct cancer is the highest among males and ranks second or third among females [7–11]. According to the latest data, which is from 2016 to 2018, the incidence of liver and bile duct cancer is the highest among males at 33.2 per 100,000 per year and the third highest among females at 12.2 per 100,000 per year. Analysis of the data categorized by region and geography revealed that liver and bile duct malignancies were more prevalent in the northeast [7].

A prior Thai study identified *Opisthorchis viverrini* infection as the primary risk factor for CCA [12]. An additional study demonstrated that CCA was present in regions affected by *O*. *viverrini* infections. The current study’s findings confirm the results of laboratory experiments in hamsters and the factors found linked to the development of CCA [13–22]. CCA has a prolonged incubation period following *O*. *viverrini* infection. Patients with CCA may show no symptoms until jaundice or hepatomegaly appear, indicating severe disease, and those with advanced CCA typically die within 3–6 months of diagnosis [1, 23–25]. In addition, the CCA predominantly affects individuals aged 35–64 [2], with a higher incidence observed in males than females [26, 27].

Spatial determinants, including proximity residing close to a river or water source, elevation, population density, and climatic variables, can elevate the risk of developing CCA. These factors can influence both the distribution of the intermediate host of *O*.*viverrini* and human exposure patterns [28, 29]. Other risk factors include the use of praziquantel for liver fluke infection, alcohol consumption, and genetic polymorphisms [30–33].

However, the complex interplay between these spatial variables and CCA incidence has not been comprehensively examined across Thailand’s diverse regions. Therefore, the purpose of the study was to understand the distribution of CCA in various regions of Thailand and identify spatial factors associated with the occurrence of CCA by the Integrating Geographic Information System technology. Also, understanding these spatial relationships is crucial for developing targeted public health strategies and informing CCA management and prevention efforts. Uncovering the spatial dynamics of CCA in Thailand can contribute valuable information for policymakers and healthcare professionals in their fight against this devastating disease.

## Material and methods

### Study design

The study was an analytical retrospective cohort study.

### Study area

The research investigated the distribution of CCA across 554 sub-districts in four regions in Thailand. The study areas, with their respective sizes and geographical coordinates, were as follows:

- Northern region (12,533.96 km^2^, 17.2°-19.5°N, 98.9°-100.2°E): Lampang Province
- Central region (6,208.70 km^2^, 14.6°-15.8°N, 100.3°-101.5°E): Lop Buri Province
- Northeastern region (10,885.99 km^2^, 15.6°-17.1°N, 101.6°-103.3°E): Khon Kaen Province
- Southern region (12,891.4 km^2^, 8.3°-10.2°N, 98.5°-100.2°E): Surat Thani Province

These coordinates encompass the latitudes and longitudes of each region, providing a comprehensive geographical context for the study.

### Study participants

Demographic data were collected from the population-based cancer registry of four institutions: Lampang Cancer Hospital in Lampang Province (northern region), Lop Buri Cancer Hospital in Lop Buri Province (central region), Khon Kaen Provincial Cancer Registry at Srinagarind Hospital, Faculty of Medicine, Khon Kaen University in Khon Kaen Province (northeastern region), and Surat Thani Cancer Hospital in Surat Thani Province (southern region) [7].

### Case definitions

The study participants were patients diagnosed with CCA residing in four regions between January 1, 2012, and December 31, 2021. The diagnosis was based on the International Classification of Diseases for Oncology, 3^rd^ Edition (ICD-O-3), with the specific codes: C22.1 (Intrahepatic bile duct), C24.0 (Extrahepatic bile duct), C24.8 (Overlapping lesion of biliary), and C24.9 (Biliary tract, NOS) (excluding C24.1, Ampulla of Vater) [34, 35].

### Population at risk

The database was retrieved for all populations residing in the sub-districts of the four regions used to calculate the ASR by sex and age groups every five years between 2012 and 2021. Data were obtained from the Office of the National Economic and Social Development Board [36].

### Power of test

This study included a sample size of all CCA cases (n = 6,379). The sample size was adequate, ensuring the study had an acceptable statistical power of more than 99% [37].

### Spatial variables

The environmental variables consisted of elevation coordinates, water source coordinates, and the size and extent of areas at the sub-district level in all four regions of Thailand. The data were obtained from the Central Geoinformatics System and Services Project, Department of Water Resources, Ministry of Natural Resources and Environment [38].

The climatic variables included average rainfall, average temperature, and a list of names and coordinates for all meteorological stations at the sub-district level of all four regions in Thailand. These data were obtained from the Thailand Meteorological Department using a statistical data request system [39].

### Data quality

The percentage of microscopically verified cases was used to determine data quality in population-based cancer registries. Upon examining the registration forms of patients with CCA in each registry, the site and sex were calculated. The percentage of microscopically verified cases values were tabulated according to site and sex, which all cases was then compared with standards established by the International Association of Cancer Registries to gauge the overall data quality [40].

### Statistical analysis

#### Descriptive epidemiology of study patients

Patient characteristics, including sex, age, and CCA cases, were summarized using descriptive statistics. Continuous variables were described using mean, standard deviation, median, and range (minimum and maximum values), whereas categorical variables were presented using frequency counts and percentages.

#### Incidence analyses

The ASRs were calculated for each sex and standardized using Segi World standard population estimates [41]. ASRs were presented as cases per 100,000 (person-years) and 95% confidence interval (CI), categorized by the period of diagnosis, sex, and age at diagnosis as per the International Association of Cancer Registries guidelines [42]. The population denominator for the ASRs was calculated using the sub-district population at risk in each region between 2012 and 2021. These populations were classified every five years based on age and sex [36].

#### Spatial pattern

This study used Moran’s *I* and Local Indicators of Spatial Association (LISA) techniques. These techniques analyze CCA risk across sub-districts and their surroundings, overcoming limitations of small areas and zero-case sub-districts in regions by considering neighboring areas collectively, thus providing a comprehensive risk assessment across the entire map.

Spatial distribution was analyzed to assess the relationship between spatial variables and the ASRs of CCA using Moran’s *I* statistic. This statistic quantifies the spatial autocorrelation by examining the spatial data observed in pairs of CCA. The value typically falls between the range of -1 to +1. Positive spatial autocorrelation occurs when neighboring places exhibit comparable values, leading to a positive *I* value. If neighboring areas have contrasting values, Moran’s *I* will be negative, indicating negative spatial autocorrelation. In a random spatial pattern, *I* is close to zero. Statistical significance was set at p < 0.05 [43].

The Anselin Local Moran’s *I*, also known as LISA, illustrates a linear relationship between two variables and is represented as a slope on a Moran scatter plot. The plot was then divided into four quadrants based on the mean values of the variable and its lagged values: high–high, high–low, low–high, and low-low [44]. This categorization aids in detecting spatial clusters and outliers by determining if adjacent sites display comparable or contrasting data.

#### Log-linear regression

Univariable analyses were performed to determine the associations of spatial variables with CCA without controlling for confounding variables. Crude incidence rate ratios (crude IRRs) were computed using univariable log-linear regression. The initial model used for multivariable analysis found a p-value of less than 0.25, indicating the significance of including spatial variables in determining the association with CCA while accounting for confounding variables.

The magnitudes of the associations obtained from multivariable analyses were represented by adjusted incidence rate ratios (adjusted IRRs), with 95%CI. A backward stepwise elimination method was used as the model fitting strategy. The best model with a low Akaike Information Criterion value was used to assess the goodness-of-fit of the final model [45].

### Coverage of cancer registries

In a previous study, a virtual three-source (clinical, morphology, and mortality) capture-recapture technique was used to determine the coverage of community cancer registries. By analyzing data from the registry, the study found that the percentage of completeness of available cases ranged from 70.0 to 99.6 percent [46].

### Data management

Variables from various sources were evaluated for accuracy, potential duplications, or omissions. We used the address code of the sub-districts as the key to connect the spatial variables within the shapefiles. Spatial and demographic variables such as the ASR of CCA, province names, province boundaries, sub-district names, sub-district boundaries, elevation, distance from water sources, population density, average rainfall, and average temperature were imported and converted into coordinates using WGS84 UTM ZONE 48 N. This data is in polyline and polygon format.

This study included descriptive epidemiological, incidence analyses, spatial patterns, and spatial analyses using R software (version 4.2.1; R Core Team) [47] with R Studio software version 1.4.1 (R) [48].

### Ethical considerations

The Human Research Ethics Committees approved the research proposal of Khon Kaen University (No. HE671027), Lampang Cancer Hospital (No. 10/2567), Lop Buri Cancer Hospital (No. LEC 6647), and Surat Thani Cancer Hospital (No. SCH_EC_01/2567). This study involved secondary data and did not involve individually identifiable information during or after data collection; therefore, informed consent was not required.

## Results

### Demographic characteristics

Between 2012 and 2021, Thailand had 6,379 CCA cases. This study included more men (n = 4,075; 63.9%) than women (n = 2,304; 36.1%). The mean age of CCA was 66.2 years (standard deviation = 11.07 years), and the majority were aged greater than 60 years (n = 4,652, 72.9%). Most of the CCA patients were in the northeastern region (Khon Kaen) (n = 3,898, 61.1%), followed by the northern region (Lampang) (n = 1,695, 26.6%), the central region (Lob Buri) (n = 624, 9.8%), and the southern region (Surat Thani) (n = 162, 2.5%) (Table 1).

**Table 1:** Demographic characteristics of CCA in Thailand between 2012 and 2021 (n = 6,379).

Characteristics	Number	Percentage
Gender		
Male	4,075	63.9
Female	2,304	36.1
Age of diagnosis (years)		
15–19	2	0.1
20–24	6	0.1
25–29	7	0.1
30–34	12	0.2
35–39	42	0.6
40–44	121	1.9
45–49	273	4.3
50–54	464	7.3
55–59	800	12.5
60–64	1,017	15.9
65–69	1,064	16.7
70–74	1,027	16.1
75+	1,544	24.2
Mean (standard deviation)	66.2 (11.07)	
Median (Minimum: Maximum)	67.0 (19: 98)	
Regions of Thailand		
Northern (Lampang)	1,695	26.6
Center (Lop Buri)	624	9.8
Northeastern (Khon Kaen)	3,898	61.1
Southern (Surat Thani)	162	2.5

### Percentage of cases microscopically verified

In Thailand, the percentage of cases microscopically verified of CCA combined with sex was higher in females (n = 532, 23.1%) than in males (n = 881, 21.6%). In terms of region, the highest percentage of cases microscopically verified was in the central region (Lob Buri) (male: n = 156, 40.7%; female: n = 105, 43.6%) and the lowest in the northeastern region (Khon Kaen) (male: n = 356, 14.0%; female: n = 190, 14.0%) (Table 2).

**Table 2:** Percentage of cases with the percentage of cases microscopically verified and incidence of CCA per 100,000 population combined by sex in each region of Thailand between 2012 and 2021.

Region	Male					Female					Both				
	Number	MV case	MV%	ASRs	95%CI	Number	MV case	MV%	ASRs	95%CI	Number	MV case	MV%	ASRs	95%CI
Northern	1,060	347	32.7	14.7	13.8 to 15.6	635	219	34.5	7.9	7.3 to 8.6	1,695	566	33.4	11.2	10.6 to 11.7
Center	383	156	40.7	6.6	5.9 to 7.3	241	105	43.6	3.4	2.9 to 3.8	624	261	41.8	4.8	4.5 to 5.2
Northeastern	2,537	356	14.0	19.2	18.4 to 19.9	1,361	190	14.0	8.5	8.0 to 8.9	3,898	546	14.0	13.4	12.9 to 13.8
Southern	95	22	23.2	1.5	1.2 to 1.8	67	18	26.9	0.8	0.6 to 1.0	162	40	24.7	1.1	0.9 to 1.3
Thailand	4,075	881	21.6	12.5	12.1 to 12.9	2,304	532	23.1	5.9	5.6 to 6.1	6,379	1,413	22.2	8.9	8.7 to 9.2

MV: morphologically verified; CI: confidence interval; ASRs: age-standardize rates.

### ASRs of CCA

In Thailand, the respective overall ASR from 2012 to 2021 for all ages in males was 12.5 per 100,000 person-years (95%CI: 12.1 to 12.9) and 5.9 per 100,000 person-years (95%CI: 5.6 to 6.1) in females. The respective ASR for each sex was 8.9 per 100,000 person-years (95%CI: 8.7 to 9.2).

The highest ASRs in both sexes were observed in the northeastern region (Khon Kaen) at 13.4 per 100 000 person-years (95%CI: 12.9 to 13.8), followed by the northern region (Lampang) at 11.2 per 100 000 person-years (95%CI: 10.6 to 11.7). The ASRs in the central region (Lob Buri) was 4.8 per 100,000 person-years (95%CI: 4.5 to 5.2), while the lowest ASRs were recorded in the southern region (Surat Thani) at 1.1 per 100,000 person-years (95%CI: 0.9 to 1.3).

The ASRs by region revealed that the northeastern region (Khon Kaen) had the highest ASRs among males at 19.2 per 100,000 person-years (95%CI: 18.4 to 19.9), followed by the northern region (Lampang) with an ASRs of 14.7 per 100,000 person-years (95%CI: 13.8 to 15.6). The central region (Lob Buri) had an ASR of 6.6 per 100,000 person-years (95%CI: 5.9 to 7.3), while the southern region (Surat Thani) had the lowest ASRs at 1.5 per 100,000 person-years (95%CI: 1.2 to 1.8).

The ASRs of females in each region revealed that the northeastern region (Khon Kaen) had the highest ASRs, with 8.5 per 100,000 person-years (95%CI: 8.0 to 8.9), followed by the northern region (Lampang) with an ASRs of 7.9 per 100,000 person-years (95%CI: 7.3 to 8.6). The central region (Lob Buri) had an ASRs of 3.4 per 100,000 person-years (95%CI: 2.9 to 3.8), while the southern region (Surat Thani) had the lowest ASRs at 0.8 per 100,000 person-years (95%CI: 0.6 to 1.0) (Table 2).

### Spatial distribution

**Fig 1:**
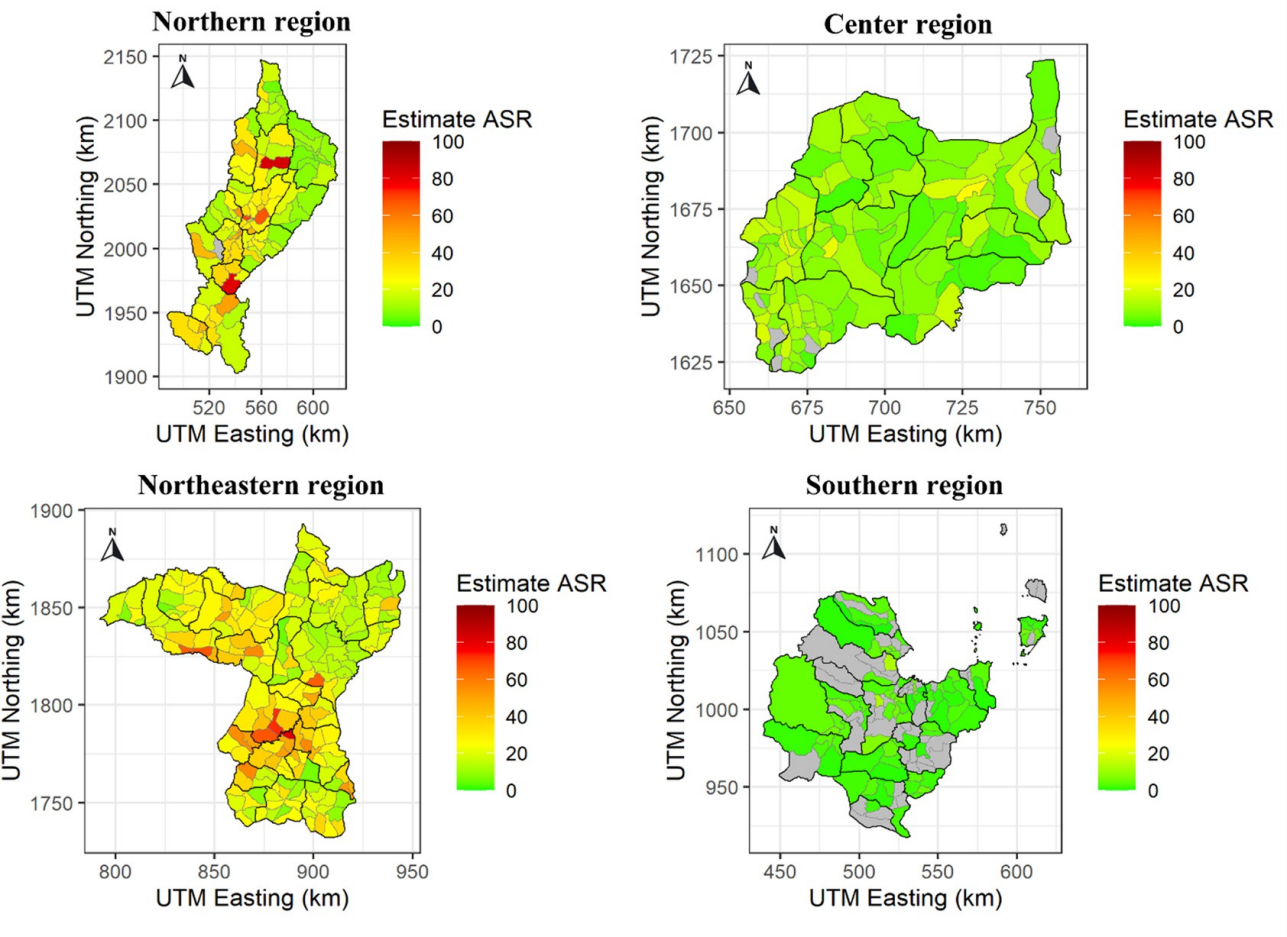
The incidence of CCA map in each region of Thailand between 2012 and 2021. https://doi.org/10.6084/m9.figshare.26196221.v1.

Fig 1 illustrates Thailand’s ASRs of CCA per 100,000 person-years at the subdistrict level for each area from 2012 to 2021. The color scheme emphasizes the geographical variation in the ASRs of CCA. Green areas represent lower ASRs, whereas dark red areas represent greater ASRs in specific sub-districts. The spatial maps show notable geographic differences in the ASRs throughout Thailand.

The highest ASRs were observed in the northern region (Lampang), particularly in the Thung Ngam Sub-district, Chae Hom Sub-district, and Sop Prap Sub-district, with ASR values of 102.4, 83.6, and 80.8, respectively. In the Central region (Lob Buri), notable areas included Lam Narai Sub-district, Wang Khon Khwang Sub-district, and Ban Kluai Sub-district exhibiting ASRs values of 25.5, 21.4, and 20.2, respectively. Within the Northeastern region (Khon Kaen), elevated ASRs values were observed in the Chonnabot district and the Wang Saeng, Suan Mon, and Non-Udom sub-districts, with values of 81.2, 72.4, and 67.2, respectively. In the Southern region (Surat Thani), ASR values were relatively lower, with notable areas including the Nam Rop, Khao Than, Taparn, and Tha Kham sub-districts, with values of 12.6, 12.6, 7.2, and 5.9, respectively (Fig 1).

### Spatial pattern

The estimated values of the Moran’s *I* statistic in Table 3 show that Moran’s *I* index of CCA and elevation variable are 0.581, 0.501, 0.242, and 0.209 in the northern region (Lampang), central region (Lob Buri), northeastern region (Khon Kaen), and southern region (Surat Thani), respectively. The positive values and p-value below 0.001 indicate substantial geographical clustering between the CCA and elevation variables. In the northern region (Lampang), the distance from the water source variable was 0.113. In the northeastern region (Khon Kaen), the population density variable was 0.453; in the southern region (Surat Thani), it was 0.111. These variables had the same positive Moran’s *I* index and significant spatial clustering as the previous variables (p-value < 0.001).

**Table 3:** Results of Moran’s *I* correlation analysis between spatial variables and CCA at sub-district level in each region of Thailand between 2012 and 2021.

Spatial variables	Northern region			Center region			Northeastern region			Southern region		
	Moran’s *I*	Z-Score	p-value	Moran’s *I*	Z-Score	p-value	Moran’s *I*	Z-Score	p-value	Moran’s *I*	Z-Score	p-value
Elevation	0.581	10.869	< 0.001	0.501	12.695	< 0.001	0.242	4.959	< 0.001	0.209	11.730	< 0.001
Distance from water sources	0.113	2.617	0.009	0.168	3.949	0.218	-0.013	-2.119	0.034	0.010	1.985	0.054
Population density	0.013	0.348	0.058	0.327	7.492	0.320	0.453	11.678	< 0.001	0.111	9.514	< 0.001
Average rainfall	-0.057	-0.868	0.386	0.071	1.943	0.052	-0.030	-0.492	0.622	-0.020	-0.605	0.5454
Average temperature	-0.050	-0.731	0.002	0.069	1.903	0.057	-0.039	-0.654	0.513	-0.029	-1.151	0.249

We identified a negative Moran’s *I* index of -0.050 for the average temperature variable in the northern region (Lampang) and -0.013 for the distance from water sources variable in the northeastern region (Khon Kaen), suggesting notable spatial dispersion, with a p-value below 0.05 (Table 3).

**Fig 2:**
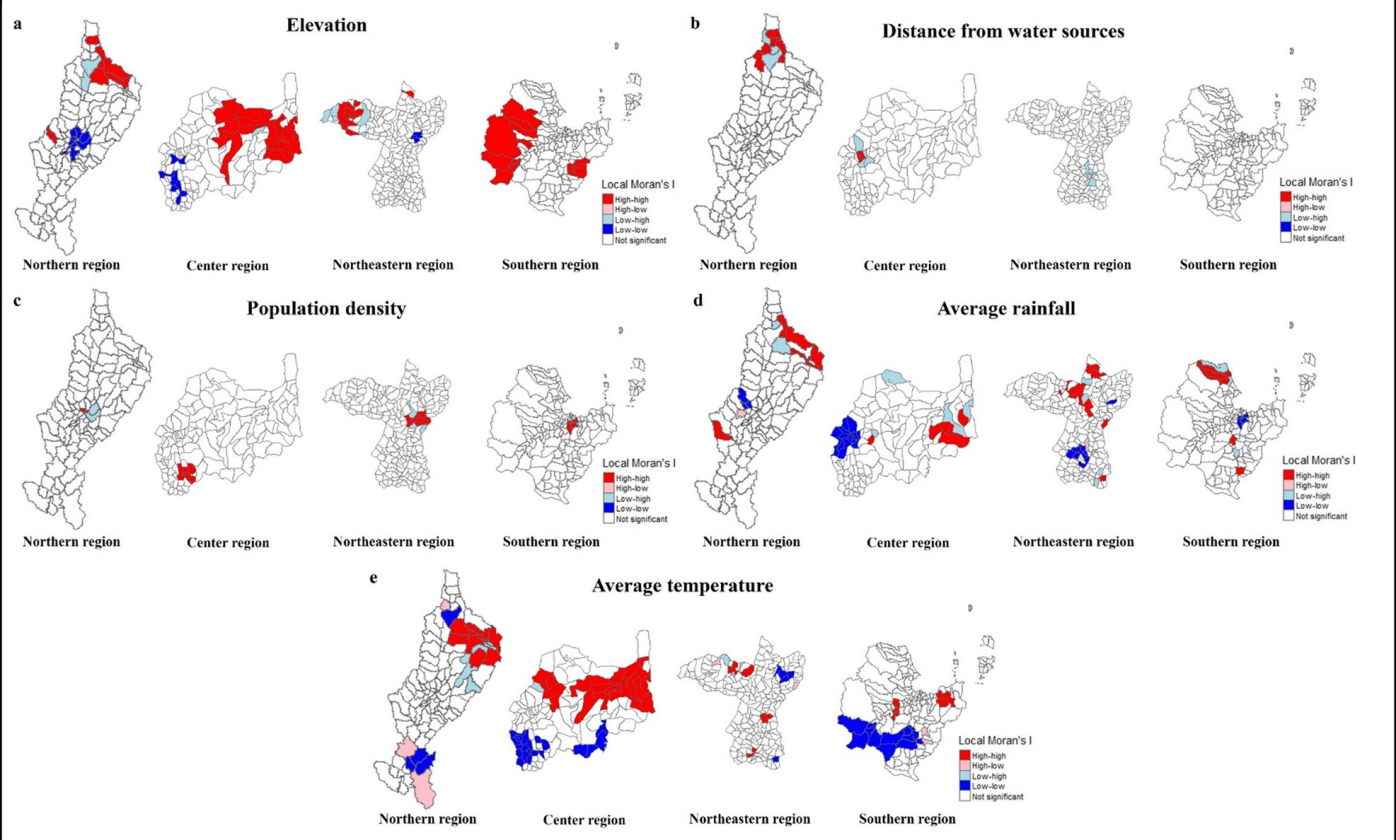
LISA analysis, incidence of CCA and spatial variables, classified by sub-district. (a) ASR of CCA with elevation variable. (b) ASR of CCA with distance from water sources. (c) ASR of CCA with population density. (d) ASR of CCA with average rainfall. (e) ASR of CCA with average temperature. https://doi.org/10.6084/m9.figshare.26196287.v1.

Table 4 and Fig 2, the LISA map shows significant positive associations at the sub-district level. High-high clusters were identified in 6 sub-districts in Lampang, 19 in Lob Buri, 15 in Khon Kaen, and 13 in Surat Thani, all related to the elevation variable. In contrast, the northern region (Lampang), central region (Lob Buri), and northeastern region (Khon Kaen) have 10, 8, and 2 sub-districts in the low-low cluster located in the region’s higher half. Our results are consistent with Moran’s I index (Fig 2A), and the high-high cluster at a distance from the water source variable is only two regions in the northern region (Lampang) in five sub-districts and the central region (Lob Buri) in three sub-districts (Fig 2B). Similarly, Fig 2C shows the population density variable in two, eight, eight, and five sub-districts in the northern region (Lampang), central region (Lob Buri), northeastern region (Khon Kaen), and southern region (Surat Thani), respectively. The clusters were in the center of the region. This outcome belongs to the high-high cluster and does not appear in the low-low cluster for this variable, akin to the distance from the water source variable (Fig 2C).

**Table 4:** Results of LISA between spatial variables and CCA in each region of Thailand between 2012 and 2021.

Spatial variables	Northern region				Center region				Northeastern region				Southern region			
	high-high	high-low	low-high	low-low	high-high	high-low	low-high	low-low	high-high	high-low	low-high	low-low	high-high	high-low	low-high	low-low
Elevation	6	-	2	10	19	-	1	8	15	-	5	2	13	-	-	-
Distance from water sources	5	-	3	-	3	-	4	-	-	2	6	-	-	-	-	-
Population density	2	-	2	-	8	-	-	-	8	-	3	-	5	-	4	-
Average rainfall	6	1	2	3	6	-	4	17	15	2	4	10	8	-	3	4
Average temperature	8	3	6	4	25	-	1	22	8	3	4	5	6	2	-	13

The average rainfall variables in the high-high cluster were the northern region (Lampang), the central region (Lob Buri), the northeastern region (Khon Kaen), and the southern region (Surat Thani) in 6, 6, 15, and 8 sub-districts, respectively. In contrast, the northern region (Lampang), central region (Lob Buri), northeastern region (Khon Kaen), and southern region (Surat Thani) in 3, 17, 10, and 4 sub-districts, respectively, belong to the low-low cluster, which is found in the upper and middle parts of the region (Fig 2D). Fig 2E shows the average temperature variable in the high-high cluster for the northern area (Lampang), central region (Lob Buri), northeastern region (Khon Kaen), and southern region (Surat Thani) in 8, 25, 8, and 6 sub-districts, respectively. The low-low cluster was found in Lampang in the north, Lob Buri in the central region, Khon Kaen in the northeast, and Surat Thani in the south, spanning 4, 22, 5, and 13 sub-districts, respectively. These clusters were identified in both the top and bottom parts of the region (Fig 2E).

### Spatial analysis

According to the results of the multivariable log-linear regression analyses in Table 5, the ASRs of CCA were negatively associated with the elevation variable in the northern region (Lampang) (adjusted IRRs = 0.82, 95%CI: 0.78 to 0.87) and the central region (Lob Buri) (adjusted IRRs = 0.85, 95%CI: 0.76 to 0.94). In addition, we found that distance from a water source in the northern region (Lampang) had an adjusted IRRs of 0.91 (95%CI: 0.82 to 0.99) compared to an adjusted IRR of 0.96 (95%CI: 0.93 to 0.99) the northeastern region (Khon Kaen), and the population density in the northeastern region (Khon Kaen) (adjusted IRRs = 0.94, 95%CI: 0.93 to 0.96) that has a negative relationship as well.

**Table 5:** Univariable and multivariable log-linear regression analyses between spatial variables and CCA in each region of Thailand between 2012 and 2021.

Spatial Variables	Northern region^1^				Center region^2^				Northeastern region^3^				Southern region^4^			
	Crude IRRs	Adjusted IRRs	95% CI	p-value	Crude IRRs	Adjusted IRRs	95% CI	p-value	Crude IRRs	Adjusted IRRs	95% CI	p-value	Crude IRRs	Adjusted IRRs	95% CI	p-value
Elevation	0.84	0.82	0.78 to 0.87	<0.001	0.88	0.85	0.76 to 0.94	0.041	1.04	-	-	-	0.91	-	-	-
Distance from water sources	0.86	0.91	0.82 to 0.99	0.031	1.00	-	-	-	0.99	0.96	0.93 to 0.99	0.001	0.61	-	-	-
Population density	0.92	-	-	-	1.01	-	-	-	0.95	0.94	0.92 to 0.96	<0.001	1.09	1.06	1.01 to 1.11	0.034
Average temperature	0.88	-	-	-	0.99	-	-	-	0.92	-	-	-	1.08	1.05	1.02 to 1.09	0.048

Crude IRRs: Crude incidence rate ratios; Adjusted IRRs: Adjusted incidence rate ratios; CI, confidence interval

^1^ After adjustment for population density, average rainfall, and average temperature

^2^ After adjustment for distance from water sources, population density, average rainfall, and average temperature

^3^ After adjustment for elevation, average rainfall, and average temperature

^4^ After adjustment for elevation, distance from water sources, and average rainfall

In addition, a different result in the southern region (Surat Thani) was positively associated with the population density variable (adjusted IRRs = 1.09, 95%CI: 1.02 to 1.15) and average temperature variable (adjusted IRRs = 1.05, 95%CI: 1.02 to 1.09) (Table 5).

## Discussion

This study aimed to investigate the association between spatial variables and CCA, including spatial variables such as the environment and climate. The results showed that most patients with CCA were older than 60 years, mainly in the northeastern (Khon Kaen) and northern regions (Lampang). In addition, this study found that areas with different elevations, residential areas with different distances from water sources, population density in each region, and average temperature in each region were associated with CCA development.

### Incidence of CCA

#### Regions of Thailand

The ASRs of CCA rate per 100,000 person-years between 2012 and 2021 in the current study was the highest in the northeastern region (Khon Kaen), followed by the northern region (Lampang), the central region (Lob Buri), and the southern region (Surat Thani) is the lowest ASR. This study is consistent with previous studies that found that males have a higher ASR than females [22]. Our study was higher than that reported in previous studies conducted with the global ASR of liver and bile duct cancer was 8.6 in both sexes, 12.7 in males and 4.8 in females [49]. In contrast, this study found that the ASR of CCA was less than the report from GLOBOCAN 2020 that found liver and bile duct cancer in Thailand in males at 33.8 and 12.9 in females [6], and the latest report in 2022 found 34.4 in males and 12.6 in females [49]. In addition, the ASR by the National Cancer Institute (NCI), the book Cancer in Thailand vol VI to X, found 42.8, 40.3, 33.9, 33.9, 33.2, and 18.2, 16.6, 12.9, 12.9, and 12.2, respectively [7–11].

The northeastern region had the highest ASR at 85.0, followed by the northern region at 14.6, the central region at 14.4, and the southern region at 5.7, according to prior studies [29, 50]. Between 1989 and 2018, prior research conducted in Khon Kaen Province reported an ASR of 36.1 (95%CI; 35.3 to 36.8) in males and 14.4 (95%CI; 13.9 to 14.8) in females. The ASRs varied by year of diagnosis. In 2002, the highest incidence was 60.2 (95%CI: 54.6 to 65.9) in male patients and 23.4 (95%CI: 20.2 to 26.6) in female patients [51]. From 1989 to 2013, the ASRs were 41.5 in males (95%CI: 40.6 to 42.4), 16.6 in females (95%CI: 16.1 to 17.7), and 28.1 for both sexes (95%CI: 27.6 to 28.7) [52]. In a study conducted in Taiwan, the incidence rate of CCA in males was 1.51 during 1993–1997, which was lower than the incidence rate of 1.73 in females. Between 2013 and 2017, the male incidence rose to 4.07, surpassing the female incidence of 2.95 [53]. The results of our study are consistent with and support prior research findings on the factors affecting CCA occurrence, such as age and gender, in each region.

### Demographic risk factors

In the current study, we found that CCA cases were related to demographic variables in patients older than 60 years, and we found that the most common cases were males. This is consistent with previous studies that found more in the age range of CCA among males than females [29, 54]. Another study found that people aged over 60 years were 2.94 times more likely to have CCA than those aged 60 years (95%CrI: 2.62 to 3.31), and males were 2.53 times more likely to have CCA compared to females (95%CrI: 2.24 to 2.85) [26]. Moreover, previous studies suggested that the male-to-female ratio is 2:1, with a mean age of 60–62 years [14, 52]. In addition, one previous study found that older individuals (50+) had a 9.21 times higher risk of developing CCA than younger individuals (95%CI: 1.10 to 77.11) [12, 13].

### Spatial risk factors

Our study focuses on the sub-district level in each region of Thailand. Our findings reveal a CCA distribution cluster in the upper and middle parts of the region. The resulting pattern of high incidence of CCA has been linked to dietary habits related to consuming raw freshwater fish, which leads to liver fluke infection factors contributing to the development of CCA. These dietary habits are often observed near water sources [29, 55]. Moreover, previous studies have reported a higher incidence of CCA in areas near water sources. Most of the population is involved in agricultural activities, such as farming or fishing, and this observation is consistent with our study [26, 29, 45, 54, 56].

A prior study focused on CCA in Laos, a landlocked country. The study findings indicated that the prevalence of *O*. *viverrini* infection was 61.1% (95%CI: 59.5 to 62.8). The study revealed that regions at higher altitudes had lower infection rates, whereas locations close to water sources had higher infection rates (odds ratio (OR) = 0.80, 95%CI: 0.67 to 0.97) [57]. Furthermore, a study conducted in Thailand found that the variable showed an inverse relationship between the prevalence of *O*. *viverrini* infection and the incidence of CCA in residential areas at higher altitudes. The prevalence of *O*. *viverrini* infection is lower in the upper area compared to the lower area [58, 59]. The results align with our investigation of residential areas at higher elevations, showing a lower ASR of CCA compared to lower regions.

Moreover, the mean night surface temperature variable (OR = 0.65, 95%CI: 0.19 to 2.27) and mean rainfall during the dry season variable remained significant after spatial random effects (OR = 0.78, 95%CI:0.54 to 1.11) [57]. The results of our study are consistent with those of other studies, where the average temperature and average rainfall variables are related to the ASR of CCA in the northern, northeastern, and southern regions.

### Data quality

The current study found that the percentage of cases microscopically verified of CCA by sex in the current study was 21.6% (n = 881) in males and 23.1% (n = 532) in females. The highest rate was observed in the central region (Lop Buri), followed by the northern region (Lampang), the southern region (Surat Thani), and the northeastern region (Khon Kaen). The report from “Cancer in Thailand Vol. VI to X” states that the average percentage of cases microscopically verified for liver and bile duct cancer is 18.6% in males (total cases = 14,950, range = 9.2% to 32.0%) and 22.3% in females (total cases = 6279, range = 10.7% to 37.3%). The highest rates were found in the Southern region (Surat Thani), the Central region (Lop Buri), the Northern region (Lampang), and the Northeastern region (Khon Kaen) [7–11]—the latest version of Cancer Incidence in Five Continents (CIV) Vol. XII and the IARC reported on liver cancer and CCA in Thailand. The study found prevalence rates in different regions: the northern region (Lampang) had rates of 17.4% in males and 21.5% in females, the central region (Lop Buri) had rates of 16.5% in males and 20.7% in females, and the northeastern region (Khon Kaen) had rates of 10.1% in males and 10.8% in females. The present study had a greater percentage compared to prior reports [40].

### Strengths of the study

First, the data used in our study were obtained from a population-based Cancer Registry in four regions. Second, our study was conducted on a large scale, which allowed us to have a representative population of individuals at risk for CCA. Additionally, we could identify the number of CCA cases by incorporating data spanning ten years. Third, this study is the first to report on spatial variable analysis at the sub-district level in Thailand using data covering the entire country. Finally, our analysis of the address code allows a more precise understanding of which neighborhoods have a higher case burden than analysis at spatial scales.

### Limitations of the study

The first limitation of this study is that we focused on spatial variables and not the causative framework, which lacks variables such as *O*. *viverrini* infection, the use of praziquantel for the treatment of *O*. *viverrini* infection, hepatitis B infection, drinking alcoholic beverages, smoking, and family history of cancer, which is critical in the development of CCA. Second, this study was geographically limited. Although it was conducted in four regions of Thailand, each region is not area contiguous, making it impossible to analyze the country using some statistics. Finally, the percentage of cases microscopically verified of CCA was low in some regions. We recommend using multiple imputations to classify the unknowns because significant histological data for CCA is missing.

## Conclusions

We demonstrated the spatial variables associated with CCA. The ASR varies by region in Thailand. Policymakers should emphasize the potential environment and climate for CCA prevention and control throughout Thailand.
